# The atypical neuroleptics iloperidone and lurasidone inhibit human cytochrome P450 enzymes in vitro. Evaluation of potential metabolic interactions

**DOI:** 10.1007/s43440-020-00102-5

**Published:** 2020-04-11

**Authors:** Przemysław J. Danek, Jacek Wójcikowski, Władysława A. Daniel

**Affiliations:** Department of Pharmacokinetics and Drug Metabolism, Maj Institute of Pharmacology, Polish Academy of Sciences, Smętna 12, 31-343 Kraków, Poland

**Keywords:** Iloperidone, Lurasidone, Cytochrome P450, Inhibition, Human liver microsomes, cDNA-expressed CYP enzymes

## Abstract

**Background:**

The present study aimed at examining the inhibitory effect of two atypical neuroleptics iloperidone and lurasidone on the main human cytochrome P450 (CYP) enzymes in pooled human liver microsomes and cDNA-expressed CYP enzymes (supersomes).

**Methods:**

The activity of these enzymes was determined by the following CYP-specific reactions: caffeine 3-*N*-demethylation/CYP1A2, diclofenac 4′-hydroxylation/CYP2C9, perazine *N*-demethylation/CYP2C19, bufuralol 1′-hydroxylation/CYP2D6 and testosterone 6β-hydroxylation/CYP3A4, respectively, using HPLC.

**Results:**

Iloperidone inhibited the activity of CYP3A4 via a noncompetitive mechanism (*K*_i_ = 0.38 and 0.3 µM in liver microsomes and supersomes, respectively) and CYP2D6 via a competitive mechanism (*K*_i_ = 2.9 and 10 µM in microsomes and supersomes). Moreover, iloperidone attenuated the activity of CYP1A2 (*K*_i_ = 45 and 31 µM in microsomes and supersomes) and CYP2C19 via a mixed mechanism (*K*_i_ = 6.5 and 32 µM in microsomes and supersomes) but did not affect CYP2C9. Lurasidone moderately inhibited CYP1A2 (*K*_i_ = 12.6 and 15.5 µM in microsomes and supersomes), CYP2C9 (*K*_i_ = 18 and 3.5 µM in microsomes and supersomes) and CYP2C19 via a mixed mechanism (*K*_i_ = 18 and 18.4 µM in microsomes and supersomes), and CYP3A4 via a competitive mechanism (*K*_i_ = 29.4 and 9.1 µM in microsomes and supersomes). Moreover, lurasidone competitively, though weakly diminished the CYP2D6 activity (*K*_i_ = 37.5 and 85 µM in microsomes and supersomes).

**Conclusion:**

The examined neuroleptics showed inhibitory effects on different CYP enzymes. The obtained results indicate that metabolic/pharmacokinetic interactions with iloperidone (involving mainly CYP3A4 and CYP2D6) and possibly with lurasidone (involving CYP1A2, CYP2C9 or CYP2C19) may occur during combined therapy.

## Introduction

The cytochromes P450 (CYPs) constitute the major family of enzymes capable of catalyzing the oxidative biotransformation of most drugs and other lipophilic xenobiotics, therefore, they are of particular relevance to clinical pharmacology [1]. CYPs are phase I enzymes that play a major role in the metabolism of endogenous compounds (e.g. steroids, monoaminergic neurotransmitters, arachidonic acid, vitamins), environmental toxins and dietary substances. The CYP family members CYP1–CYP3 are responsible for the biotransformation of most foreign substances, including 70–80% of all drugs in clinical use [2, 3].

Iloperidone, a piperidinyl benzisoxazole derivative, is an atypical neuroleptic drug approved for the treatment of acute schizophrenia in adult patients [4]. It produces an antagonistic effect by a high-affinity binding to serotonin 5-HT_2A_, and dopamine D_2_ and D_3_ receptors, moderate affinity binding to dopamine D_4_, serotonin 5-HT_6_ and 5-HT_7_, and norepinephrine α_1_ receptors, and low-affinity binding to serotonin 5-HT_1A_, dopamine D_1_, and histamine H_1_ receptors. Iloperidone has no appreciable affinity for muscarinic receptors [5, 6]. The drug is mainly metabolized by CYP2D6, and to a lesser degree by CYP3A4 [7] to two major metabolites: P88-8991 and P95-12113. CYP2D6 catalyzes its hydroxylation to metabolite P94, which can undergo further metabolism to P95, while CYP3A4 catalyzes *O*-demethylation to generate P89 and undergo carbonyl reduction to metabolite P88 [4, 8, 9].

Lurasidone, an azapirone derivative with a benzisothiazol-piperazine side chain [10], is also an atypical neuroleptic indicated for the treatment of schizophrenia and depressive episodes associated with bipolar I disorder, as monotherapy or adjunctive treatment with lithium or valproate [11]. This atypical neuroleptic drug has a strong antagonistic action at D_2,_ 5-HT_2A_ and 5-HT_7_ receptors. It also has a high affinity as a partial agonist at the 5-HT_1A_ receptors, and a moderate affinity for adrenergic receptors α_2A_ and α_2C_ [6, 12]. The drug has no action on histaminergic and muscarinic receptors. Lurasidone is primarily metabolized in the liver by CYP3A4 [13]. Its major biotransformation pathways include oxidative *N*-dealkylation, hydroxylation of norbornane ring or cyclohexane ring, *S*-oxidation, reductive cleavage of the isothiazole ring, followed by *S*-methylation or combinations of two or more of these pathways [11]. Lurasidone is broken down to three active metabolites (ID-14283, ID-14326 and ID-14614) and two inactive metabolites (ID-20219 and its hydroxylated derivative ID-20220) [14].

Among atypical antipsychotic agents, each has a distinctive pharmacokinetic profile including a unique metabolism and excretion pathway. The metabolic pathways engaging particular CYP-enzymes dictate the likelihood that an agent will participate in cytochrome P450-mediated pharmacokinetic drug-drug interactions. Metabolic interactions may proceed via enzyme inhibition or induction, or competition for a specific hepatic CYP enzyme [9]. The potential for this kind of interactions is the highest when concomitantly administered drugs are metabolized by the same CYP enzyme. In addition, many compounds can also be strong inhibitors of CYP enzymes, not directly involved in their clearance, but can greatly affect the metabolism of other coadministered drugs [15].

According to scientific literature data, possible drug interactions with the atypical neuroleptics iloperidone and lurasidone at a level of cytochrome P450 are not well known. Therefore, the purpose of the present study was to investigate possible inhibitory effects of the above-mentioned drugs on the main CYP enzymes of human liver: CYP1A2, CYP2C9, CYP2C19, CYP2D6 and CYP3A4.

## Materials and methods

### Drugs and chemicals

Lurasidone and iloperidone were obtained from TargetMol (Boston, USA). Caffeine, 3-*N*-desmethyl caffeine (paraxanthine), diclofenac, 4′-hydroxydiclofenac, bufuralol, 1′-hydroxybufuralol, NADP, NADPH, glucose-6-phosphate, glucose-6-phosphate-dehydrogenase, MgCl_2_, KCl, ZnSO_4_, Trizma base and ethylenediaminetetraacetic acid (EDTA) were purchased from Sigma (St. Louis, USA). Testosterone and 6β-testosterone were from Steraloids (Newport, USA). All the organic solvents with HPLC purity were supplied by Merck (Darmstadt, Germany). Pooled human liver microsomes and microsomes from baculovirus-infected insect cells expressing human CYP1A2, CYP2C9, CYP2C19, CYP2D6 and CYP3A4 (supersomes) were provided by Corning (Woburn, USA).

### Determination of CYP enzyme activities

To study the inhibitory effects of iloperidone and lurasidone on the activity of various CYP isoforms, pooled human liver microsomes and microsomes from baculovirus-infected insect cells expressing human CYPs (supersomes) were used. The following probe reactions were applied, according to the methods previously described [16–21]: caffeine 3-*N*-demethylation for CYP1A2 (caffeine 200, 400 and 800 µM), diclofenac 4′-hydroxylation for CYP2C9 (diclofenac 5, 10, 25 µM), perazine *N*-demethylation for CYP2C19 (perazine 50, 100, 200 µM), bufuralol 1′-hydroxylation for CYP2D6 (bufuralol 10, 25, 50 µM), and testosterone 6β-hydroxylation for CYP3A4 (50, 100 and 200 µM). Incubation systems for CYP2C9, 2C19 and 3A4 contained: 50 mM TRIS/KCL buffer (pH = 7.4), NADPH generating system (1 mM NADP, 5 mM glucose 6-phosphate, 1.7 U/ml glucose 6-phosphate dehydrogenase, 1 mM EDTA and 3 mM MgCl_2_). Incubation mixture for CYP1A2 included: 0.15 M phosphate buffer (pH = 7.4) and 1 mM NADPH, and for CYP2D6: 0.1 M TRIS/KCL buffer (pH = 7.4), NADPH generating system (1.3 mM NADP, 3.3 mM glucose 6-phosphate, 1 U/ml glucose 6-phosphate dehydrogenase and 3.3 mM MgCl_2_). The appropriate concentrations of human liver microsomes (0.5 mg/ml for each reaction) or supersomes (50 pmol CYP/ml), various concentrations of a probe substrate in the absence or presence of neuroleptic (concentrations: 0.1, 0.5, 1, 5, 10 µM) were added, The final volume of the reaction mixture was 0.5 ml. The incubation time for supersomes was 30 min (for each reaction) and for liver microsomes: 30 min (diclofenac 4′-hydroxylation and bufuralol 1′-hydroxylation), 20 min (perazine *N*-demethylation and testosterone 6β-hydroxylation) or 50 min (caffeine 3-*N*-demethylation). After the reactions had been stopped, the concentrations of specific substrates and their metabolites formed in liver microsomes or supersomes were assessed by the HPLC method with UV detection (or fluorimetric detection for CYP2D6), as described previously [16–21].

### *Determination of kinetic parameters, K*_*i*_* values and the mechanism of inhibition*

Kinetic parameters (*K*_m_, *V*_max_, *K*_i_) describing the course of CYP-specific reactions in liver microsomes or supersomes were obtained using the Michaelis–Menten approach and a non-linear regression analysis (Program Sigma Plot 12.3 Enzyme Kinetics). The inhibitory effects of iloperidone and lurasidone on CYP enzymes are presented graphically as Dixon’s plots (1/*V* against I) indicating *K*_i_ values, and Lineweaver–Burk’s plots (1/*V* against 1/*S*) showing the mechanism of inhibition (competitive inhibition increases the *K*_m_ value, not affecting the *V*_max_ value; non-competitive inhibition decreases the *V*_max_ value, not affecting the *K*_m_ value; mixed inhibition entails respective changes in both the *K*_m_ and *V*_max_ values).

## Results

To investigate whether iloperidone and lurasidone affect the activity of CYP enzymes, the probe reaction assays were conducted with varied concentration of the neuroleptics. The Dixon`s plots of the metabolism of CYP-specific substrates, carried out in human liver microsomes and supersomes CYP1A2, CYP2D6, CYP2C9, CYP2C19 and CYP3A4, in the absence or presence of the tested neuroleptics, showed that the examined neuroleptics exerted inhibitory effects on different CYP enzymes. However, their potency to inhibit specific CYP enzymes was diverse. Iloperidone exerted a strong inhibitory effect on the activity of CYP3A4 (*K*_i_ = 0.38 and 0.3 µM in liver microsomes and supersomes, respectively) and CYP2D6 (*K*_i_ = 2.9 and 10 µM in liver microsomes and supersomes, respectively). Moreover, iloperidone attenuated the activity of CYP2C19 (*K*_i_ = 6.5 and 32 µM in liver microsomes and supersomes, respectively) and CYP1A2 (*K*_i_ = 45 and 31 µM in liver microsomes and supersomes, respectively). Iloperidone did not affect the activity of CYP2C9. In comparison, lurasidone moderately inhibited CYP1A2 (*K*_i_ = 12.6 and 15.5 µM in liver microsomes and supersomes, respectively), CYP2C9 (*K*_i_ = 18 and 3.5 µM in liver microsomes and supersomes, respectively), CYP2C19 (*K*_i_ = 18 and 18.4 µM in liver microsomes and supersomes, respectively) and CYP3A4 (*K*_i_ = 29.4 and 9.1 µM in liver microsomes and supersomes, respectively). Lurasidone weakly diminished the activity of CYP2D6 (*K*_i_ = 37.5 and 85 µM in liver microsomes and supersomes, respectively).

**Fig. 1:**
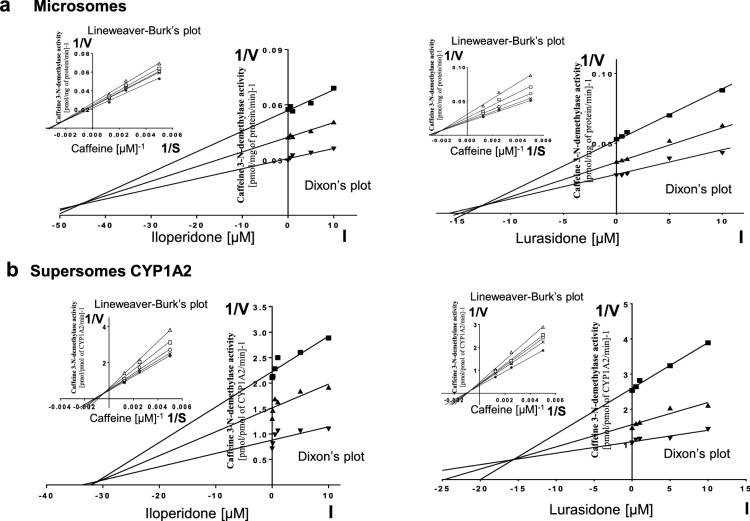
The influence of iloperidone and lurasidone on the activity of CYP1A2 measured as a rate of caffeine 3-*N-*demethylation. **a** Human liver microsomes (iloperidone: *K*_m_ = 293 µM, *V*_max_ = 64.7 pmol/mg protein/min; lurasidone: *K*_m_ = 407.6 µM, *V*_max_ = 57.96 pmol/mg protein/min). **b** Human cDNA-expressed CYP1A2 (Supersomes CYP1A2) (iloperidone: *K*_m_ = 571 µM, *V*_max_ = 3.68 pmol/mg protein/min; lurasidone: *K*_m_ = 617 µM, *V*_max_ = 1.74 pmol/mg protein/min). Each point represents the mean value of two independent analyses. *V* velocity of the reaction, *I* the concentration of the inhibitor (iloperidone or lurasidone), *S* the concentration of the substrate (caffeine). The *K*_i_ values and mechanisms of inhibition are shown in Table 1. Dixon’s plots (the main plots): the caffeine concentration of 200 µM (filled square), 400 µM (filled upwardtriangle) and 800 µM (filled downward triangle). Lineweaver–Burk’s plots (inserts): control—no inhibitor (no iloperidone or lurasidone) (asterisk); the inhibitor (iloperidone or lurasidone) concentration of 0.5 µM (cross mark), 1 µM (unfilled circle), 5 µM (unfilled square) and 10 µM (unfilled upward triangle)

**Fig. 2:**
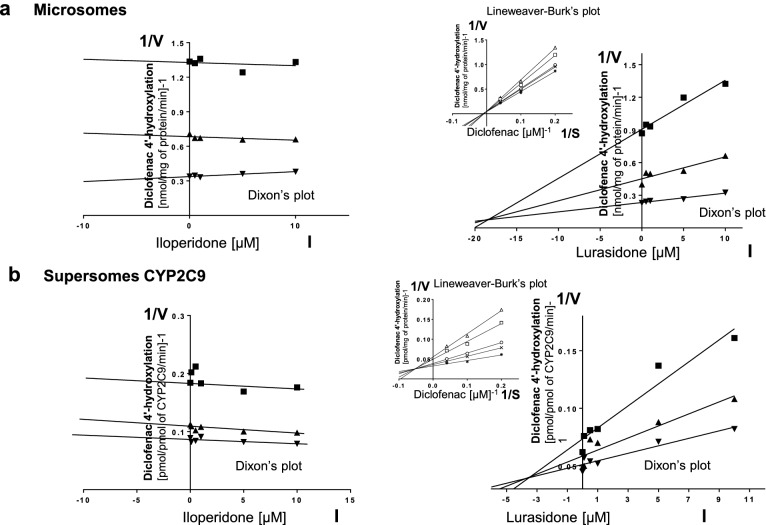
The influence of iloperidone or lurasidone on the activity of CYP2C9 measured as a rate of diclofenac 4′-hydroxylation. **a** Human liver microsomes (iloperidone: *K*_m_ = 73.13 µM, *V*_max_ = 11.64 nmol/mg protein/min; lurasidone: *K*_m_ = 95.16 µM, *V*_max_ = 16.45 nmol/mg protein/min). **b** Human cDNA-expressed CYP2C9 (supersomes CYP2C9) (iloperidone: *K*_m_ = 6.17 µM, *V*_max_ = 14.46 pmol/mg protein/min; lurasidone: *K*_m_ = 4.07 µM, *V*_max_ = 14.55 pmol/mg protein/min). Each point represents the mean value of two independent analyses. *V* velocity of the reaction, *I* the concentration of the inhibitor (iloperidone or lurasidone), *S* the concentration of the substrate (diclofenac). The *K*_i_ values and mechanisms of inhibition are shown in Table 1. Dixon’s plots (the main plots): the diclofenac concentration of 5 µM (filled square), 10 µM (filled upward triangle) and 25 µM (filled downward triangle). Lineweaver–Burk’s plots (inserts): control—no inhibitor (no lurasidone) (asterisk); the lurasidone concentration of 0.5 µM (cross mark), 1 µM (unfilled circle), 5 µM (unfilled square) and 10 µM (unfilled upward triangle)

**Fig. 3:**
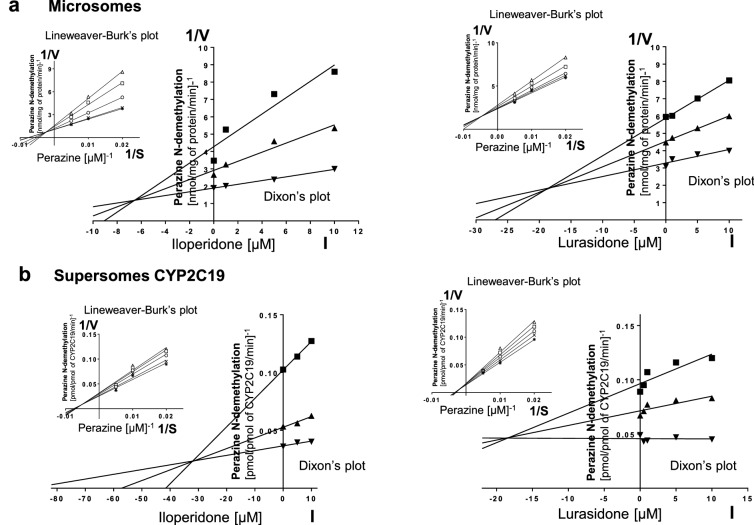
The influence of iloperidone and lurasidone on the activity of CYP2C19 measured as a rate of perazine *N*-demethylation. **a** Human liver microsomes (iloperidone: *K*_m_ = 79.69 µM, *V*_max_ = 0.76 nmol/mg protein/min; lurasidone: *K*_m_ = 71.6 µM, *V*_max_ = 0.44 nmol/mg protein/min). **b** Human cDNA-expressed CYP2C19 (supersomes CYP2C19) (iloperidone: *K*_m_ = 155.4 µM, *V*_max_ = 32.56 pmol/mg protein/min; lurasidone: *K*_m_ = 176.6 µM, *V*_max_ = 44.57 pmol/mg protein/min). Each point represents the mean value of two independent analyses. *V* velocity of the reaction, *I* the concentration of the inhibitor (asenapine), *S* the concentration of the substrate (perazine). The *K*_i_ values and mechanisms of inhibition are shown in Table 1. Dixon’s plots (the main plots): the perazine concentration of 50 µM (filled square), 100 µM (filled upward triangle) and 200 µM (filled downward triangle). Lineweaver–Burk’s plots (inserts): control – no inhibitor (no iloperidone or lurasidone) (asterisk); the inhibitor (iloperidone or lurasidone) concentration of 0.5 µM (cross mark), 1 µM (unfilled circle), 5 µM (unfilled square) and 10 µM (unfilled upward triangle)

**Fig. 4:**
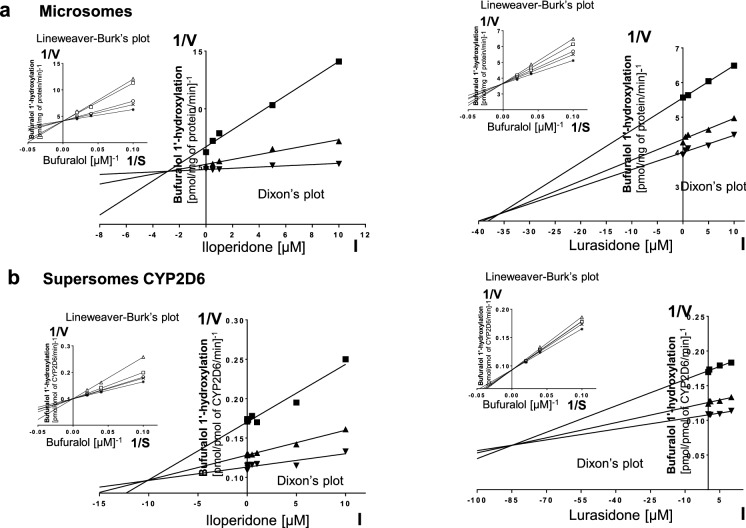
The influence of iloperidone and lurasidone on the activity of CYP2D6 measured as a rate of bufuralol 1′-hydroxylation. **a** Human liver microsomes (iloperidone: *K*_m_ = 3.84 µM, *V*_max_ = 0.23 pmol/mg protein/min; lurasidone: *K*_m_ = 5.48 µM, *V*_max_ = 0.28 pmol/mg protein/min). **b** Human cDNA-expressed CYP2D6 (supersomes CYP2D6) (iloperidone: *K*_m_ = 7.31 µM, *V*_max_ = 9.95 pmol/mg protein/min; lurasidone: *K*_m_ = 18.69 µM, *V*_max_ = 10.7 pmol/mg protein/min). Each point represents the mean value of two independent analyses. *V* velocity of the reaction, *I* the concentration of the inhibitor (iloperidone or lurasidone), *S* the concentration of the substrate (bufuralol). The *K*_i_ values and mechanisms of inhibition are shown in Table 1. Dixon’s plots (the main plots): the bufuralol concentration of 10 µM (filled square), 25 µM (filled upward triangle) and 50 µM (filled downward triangle). Lineweaver–Burk’s plots (inserts): control—no inhibitor (no iloperidone or lurasidone) (asterisk); the inhibitor (iloperidone or lurasidone) concentration of 0.5 µM (cross mark), 1 µM (unfilled circle), 5 µM (unfilled square) and 10 µM (unfilled upward triangle)

**Fig. 5:**
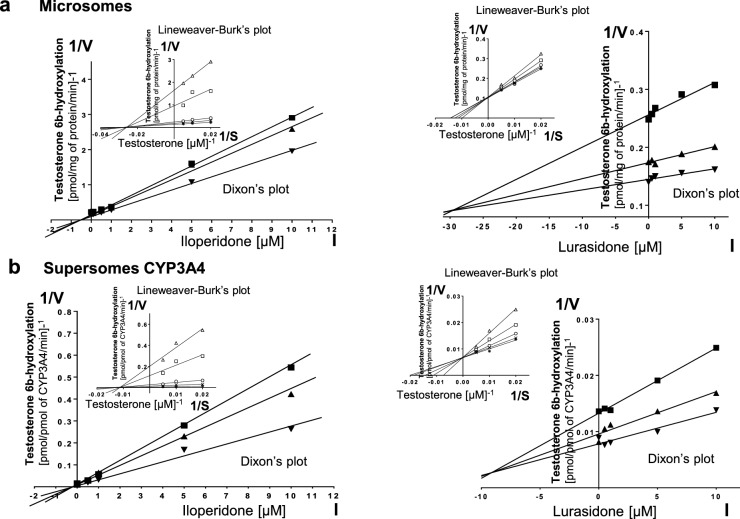
The influence of iloperidone or lurasidone on the activity of CYP3A4 measured as a rate of testosterone 6β-hydroxylation. **a** Human liver (iloperidone: *K*_m_ = 31.19 µM, *V*_max_ = 7.51 pmol/mg protein/min; lurasidone: *K*_m_ = 85.01 µM, *V*_max_ = 11.21 pmol/mg protein/min). **b** Human cDNA-expressed CYP3A4 (supersomes CYP3A4) (iloperidone: *K*_m_ = 78.7 µM, *V*_max_ = 176.3 pmol/mg protein/min; lurasidone: *K*_m_ = 58.83 µM, *V*_max_ = 155.1 pmol/mg protein/min). Each point represents the mean value of two independent analyses. *V* velocity of the reaction, *I* the concentration of the inhibitor (iloperidone or lurasidone), *S* the concentration of the substrate (testosterone). The *K*_i_ values and mechanisms of inhibition are shown in Table 1. Dixon’s plots (the main plots): the testosterone concentration of 50 µM (filled square), 100 µM (filled upward triangle) and 200 µM (filled downward triangle). Lineweaver–Burk’s plots (inserts): control—no inhibitor (no iloperidone or lurasidone) (asterisk); the inhibitor (iloperidone or lurasidone) concentration of 0.5 µM (cross mark), 1 µM (unfilled circle), 5 µM (unfilled square) and 10 µM (unfilled upward triangle)

Lineweaver–Burk’s plots referring to the kinetics of enzyme inhibition suggested that in both human liver microsomes and supersomes iloperidone inhibited the activity of CYP3A4 via a noncompetitive mechanism, CYP2D6 via a competitive mechanism, CYP1A2 and CYP2C19 via a mixed mechanism (inserts in Figs. 1, 3, 4, 5). On the other hand, lurasidone inhibited the activity of CYP1A2, CYP2C9 and CYP2C19 via a mixed mechanism, CYP3A4 and CYP2D6 via a competitive mechanism (inserts in Figs. 1, 2, 3, 4, 5. The *K*_i_ values and mechanisms of inhibition of major human CYP enzyme activities by iloperidone and lurasidone are summarized in Table 1.

**Table 1:** The ability of iloperidone and lurasidone to inhibit CYP1A2, CYP2C9, CYP2C19, CYP2D6 and CYP3A4 activities in vitro in human liver microsomes and human cDNA-expressed CYP enzymes (supersomes)

CYPs isoenzymes	Inhibition of CYP-specific reactions by iloperidone and lurasidone *K*_i_ (µM) and type of inhibition			
	Iloperidone		Lurasidone	
	Liver microsomes	Supersomes	Liver microsomes	Supersomes
CYP1A2	45 Mixed	31 Mixed	12.6 Mixed	15.5 Mixed
CYP2C9	–	–	18 Mixed	3.5 Mixed
CYP2C19	6.5 Mixed	32 Mixed	18 Mixed	18.4 Mixed
CYP2D6	2.9 Competitive	10 Competitive	37.5 Competitive	85 Competitive
CYP3A4	0.38 Noncompetitive	0.3 Noncompetitive	29.4 Competitive	9.1 Competitive

The presented inhibition constants (*K*_i_) for the inhibition of particular CYP-specific reactions by iloperidone and lurasidone were obtained using a non-linear regression analysis (Program Sigma Plot 12.3; enzyme kinetics) and are shown graphically in Figs. 1, 2, 3, 4, 5 (Dixon’s plots). The mechanisms of inhibition were estimated on the basis of changes in the *K*_m_ and/or *V*_max_ values by the tested inhibitors (iloperidone and lurasidone) and are shown graphically in the inserts of Figs. 1, 2, 3, 4, 5 (Lineweaver–Burk’s plots)

## Discussion

Clinically significant drug-drug interactions are defined as events in which the pharmacodynamic or pharmacokinetic characteristics of a drug are modified by the addition of a second drug to the patient’s medication regimen, which results in an increase in serious adverse reactions or attenuation of efficacy [22]. Interactions may occur when concomitantly administered medications share similar targets (e.g. receptor) producing either additive or antagonistic effects that can enhance or weaken the pharmacological effect of the primary drug, respectively [23]. Clinically significant interactions of drugs acting on the central nervous system can produce extrapyramidal symptoms, depression, seizures, serotonin syndrome and many more [24]. The majority of pharmacokinetic drug-drug interactions involve alterations in phase I metabolism by inhibition or induction of cytochrome P450. CYP enzymes, in particular CYP1A2, CYP2C9, CYP2C19, CYP2D6 and CYP3A4, are responsible for most drug metabolism in humans [25].

The results of our present study show that iloperidone and lurasidone exert inhibitory effects on CYP1A2, CYP2C9 (only lurasidone), CYP2C19, CYP2D6 and CYP3A4 activities in pooled human liver microsomes and microsomes from baculovirus-infected insect cells expressing human CYPs (supersomes). However, their potency to inhibit specific CYP enzymes was diverse. Iloperidone potently inhibited CYP3A4 and CYP2D6 via a noncompetitive or competitive mechanism (respectively). Moreover, it moderately diminished the activity of CYP2C19 and weakly affected CYP1A2 via a mixed mechanism. In the case of lurasidone, moderate inhibition of CYP1A2, CYP2C9 and CYP2C19 via a mixed mechanism and of CYP3A4 via a competitive mechanism was observed. The CYP2D6 activity was weakly inhibited via a competitive mechanism.

The potent inhibition of CYP3A4 and CYP2D6 by iloperidone displays *K*_i_ values which are situated in the range of the *K*_i_ values observed for such potent CYP3A4 inhibitors as ritonavir, verapamil or erythromycin (*K*_i_ = 0.1 − 5 µM) [26], as well as in the range of the therapeutic concentration of the neuroleptic (up to 0.2 μM) [27, 28]. Hence, a potent inhibition of CYP3A4 and CYP2D6 by iloperidone observed in vitro in the present study may be expected to occur in vivo, since the *K*_i_ values calculated for human liver microsomes (0.38 and 2.9 µM, respectively) are close to the presumed concentration range of iloperidone in the liver of psychiatric patients. Since iloperidone is both a substrate and an inhibitor of CYP3A4 and CYP2D6, it seems possible that the neuroleptic can also inhibit its own metabolism. Iloperidone is also expected to moderately alter the exposure to drugs primarily cleared by CYP2C19 such as diazepam, omeprazole, mephenytoin or tricyclic antidepressants [29–32]. The magnitude of interaction may be more pronounced if alternative metabolic pathways are inhibited simultaneously. In addition, the clinical impact of iloperidone might be greater when it is co-prescribed with prodrugs requiring CYP2C19-mediated metabolic activation (e.g. clopidogrel) [33].

Although lurasidone can be categorized as a moderate inhibitor of CYP2C9, it may have clinically important consequences for some substrates with a narrow therapeutic range (e.g. warfarin, phenytoin). Similarly, the moderate inhibition of CYP1A2 or CYP2C19 by lurasidone demonstrated in vitro in the present study, may be of pharmacological significance in vivo for drugs mainly metabolized or bioactivated by the mentioned enzymes (e.g. bioactivation of antiandrogen flutamide by CYP1A2) [34].

Albeit the therapeutic plasma concentrations of iloperidone and lurasidone reach the level up to 0.2 and 1.2 μM, respectively [11, 27, 28, 35], their concentrations in the liver may be several times higher than in the plasma owing to their physicochemical properties manifesting themselves by a very high lipophilicity (log *P* = 4.43 for the iloperidone and 5.6 for the lurasidone) and related tissue distribution pattern [36–38]. Drugs, that are lipophilic in nature, are characterized by the extensive accumulation in tissues due to nonspecific binding to cellular membranes [39–41], including endoplasmic reticulum where liver cytochrome P450 is anchored.

Despite the potent inhibition of CYP2C19 and CYP2D6 in human liver microsomes (6.5 and 2.9 µM, respectively), iloperidone exerts a weaker inhibitory effect on cDNA-expressed CYP2C19 and CYP2D6 in supersomes (32 and 10 µM, respectively). A similar situation was observed for lurasidone in the case of the CYP2D6 enzyme, i.e. stronger inhibition occurred in liver microsomes (37.5 µM) than in cDNA-expressed CYP2D6 supersomes (85 µM). This kind of differences in the inhibition potency between human liver microsomes and cDNA expressed CYPs (supersomes) were also observed by Kahma et al. [42]. Zang et al. [43] proposed that the positioning of CYP enzymes in insect cell membranes may differ from their positioning on the membrane of the endoplasmic reticulum in humans. The aggregation properties of CYPs are affected by the microsomal lipid composition, the lipid/protein ratio, and the concentrations of the redox partners of CYP enzymes [44], which can differ markedly between expression systems and human cells [45]. Moreover, it should be taken into account that the penetration of substrates and inhibitors into the membranes of the endoplasmic reticulum and accumulation therein may differ between species. It may affect membrane condition, functioning of the mixed-function oxidase system (MFO) and cytochrome P450 occupation by interacting drugs. The above-described physicochemical phenomena would explain differences in the inhibitory effects observed between human microsomes and cDNA expressed CYPs (supersomes).

The knowledge of the ability of iloperidone to inhibit CYP3A4, CYP2D6 and possibility of lurasidone to inhibit CYP2C9 is of pharmacological and clinical importance, since these neuroleptics are administered to patients for months or years, and very often in combination with other clinically important drugs that are substrates of the above-mentioned CYP enzymes. Therefore, the inhibition of those CYPs by iloperidone or lurasidone may produce drug-drug interactions. The obtained results suggest that pharmacokinetic interactions involving iloperidone and substrates of CYP2D6 (e.g. tricyclic antidepressants, SSRIs, codeine, dextromethorphan, debrisoquine, metoprolol, propranolol) and CYP3A4 (e.g. antidepressants, benzodiazepines, calcium channel antagonists, macrolide antibiotics) or lurasidone and CYP2C9 substrates (e.g. warfarin, phenytoin, naproxen, diclofenac and sulfonylureas) are likely to occur in patients during co-administration of the above-mentioned drugs [31]. Given that the number of patients requiring iloperidone or lurasidone is on the rise and these patients are often seriously ill, the likelihood that the tested drugs will be co-prescribed with drugs that interact with those neuroleptics and thus elicit frequent and severe adverse drug interactions in the population is high.

## References

[1] Zanger UM, Schwab M (2013). Cytochrome P450 enzymes in drug metabolism: regulation of gene expression, enzyme activities, and impact of genetic variation. Pharmacol Ther.

[2] Wienkers LC, Heath TG (2005). Predicting in vivo drug interactions from in vitro drug discovery data. Nat Rev Drug Discov.

[3] Bibi Z (2014). Retraction: Role of cytochrome P450 in drug interactions. Nutr Metab (Lond).

[4] George M, Amrutheshwar R, Rajkumar RP, Kattimani S, Dkhar SA (2013). Newer antipsychotics and upcoming molecules for schizophrenia. Eur J Clin Pharmacol.

[5] Citrome L (2010). Iloperidone: chemistry, pharmacodynamics, pharmacokinetics and metabolism, clinical efficacy, safety and tolerability, regulatory affairs, and an opinion. Expert Opin Drug Metab Toxicol.

[6] Tarazi FI, Stahl SM (2012). Iloperidone, asenapine and lurasidone: a primer on their current status. Expert Opin Pharmacother.

[7] Wang S-M, Han C, Lee S-J, Patkar AA, Masand PS, Pae C-U (2013). Asenapine, blonanserin, iloperidone, lurasidone, and sertindole: distinctive clinical characteristics of 5 novel atypical antipsychotics. Clin Neuropharmacol.

[8] Citrome L (2009). Iloperidone for schizophrenia: a review of the efficacy and safety profile for this newly commercialised second-generation antipsychotic. Int J Clin Pract.

[9] Sheehan JJ, Sliwa JK, Amatniek JC, Grinspan A, Canuso CM (2010). Atypical antipsychotic metabolism and excretion. Curr Drug Metab.

[10] Caccia S (2011). Pharmacokinetics and metabolism update for some recent antipsychotics. Expert Opin Drug Metab Toxicol.

[11] Greenberg WM, Citrome L (2017). Pharmacokinetics and pharmacodynamics of lurasidone hydrochloride, a second-generation antipsychotic: a systematic review of the published literature. Clin Pharmacokinet.

[12] Bobo WV (2013). Asenapine, iloperidone and lurasidone: critical appraisal of the most recently approved pharmacotherapies for schizophrenia in adults. Expert Rev Clin Pharmacol.

[13] Mauri MC, Paletta S, Maffini M, Colasanti A, Dragogna F, Di Pace C (2014). Clinical pharmacology of atypical antipsychotics: an update. EXCLI J.

[14] Findling RL, Goldman R, Chiu Y-Y, Silva R, Jin F, Pikalov A (2015). Pharmacokinetics and tolerability of lurasidone in children and adolescents with psychiatric disorders. Clin Ther.

[15] Bjornsson TD, Callaghan JT, Einolf HJ, Fischer V, Gan L, Grimm S (2003). The conduct of in vitro and in vivo drug-drug interaction studies: a PhRMA perspective. J Clin Pharmacol.

[16] Daniel WA, Kot M, Wójcikowski J (2003). Influence of classic and atypical neuroleptics on caffeine oxidation in rat liver microsomes. Pol J Pharmacol.

[17] Basińska-Ziobroń A, Daniel WA, Wójcikowski J (2015). Inhibition of human cytochrome P450 isoenzymes by a phenothiazine neuroleptic levomepromazine: an in vitro study. Pharmacol Rep.

[18] Schmitz G, Lepper H, Estler C-J (1993). High-performance liquid chromatographic method for the routine determination of diclofenac and its hydroxy and methoxy metabolites from in vitro systems. J Chromatogr B Biomed Sci Appl.

[19] Wójcikowski J, Pichard-Garcia L, Maurel P, Daniel WA (2004). The metabolism of the piperazine-type phenothiazine neuroleptic perazine by the human cytochrome P-450 isoenzymes. Eur Neuropsychopharmacol.

[20] Hiroi T, Chow T, Imaoka S, Funae Y (2002). Catalytic specificity of CYP2D isoforms in rat and human. Drug Metab Dispos.

[21] Wójcikowski J, Haduch A, Daniel WA (2012). Effect of classic and atypical neuroleptics on cytochrome P450 3A (CYP3A) in rat liver. Pharmacol Rep.

[22] Leucuta SE, Vlase L (2006). Pharmacokinetics and metabolic drug interactions. Curr Clin Pharmacol.

[23] Sandson N (2005). Drug-drug interactions: the silent epidemic. Psychiatr Serv.

[24] Prior TI, Baker GB (2003). Interactions between the cytochrome P450 system and the second-generation antipsychotics. J Psychiatry Neurosci.

[25] Haduch A, Daniel WA (2018). The engagement of brain cytochrome P450 in the metabolism of endogenous neuroactive substrates: a possible role in mental disorders. Drug Metab Rev.

[26] Parmentier Y, Pothier C, Delmas A, Caradec F, Trancart M-M, Guillet F (2017). Direct and quantitative evaluation of the human CYP3A4 contribution (fm) to drug clearance using the in vitro SILENSOMES model. Xenobiotica.

[27] Hiemke C, Bergemann N, Clement HW, Conca A, Deckert J, Domschke K (2018). Consensus guidelines for therapeutic drug monitoring in neuropsychopharmacology: update 2017. Pharmacopsychiatry.

[28] Caccia S, Pasina L, Nobili A (2010). New atypical antipsychotics for schizophrenia: iloperidone. Drug Des Devel Ther.

[29] Fukasawa T, Suzuki A, Otani K (2007). Effects of genetic polymorphism of cytochrome P450 enzymes on the pharmacokinetics of benzodiazepines. J Clin Pharm Ther.

[30] Desta Z, Zhao X, Shin JG, Flockhart DA (2002). Clinical significance of the cytochrome P450 2C19 genetic polymorphism. Clin Pharmacokinet.

[31] Zanger UM, Schwab M (2013). Cytochrome P450 enzymes in drug metabolism: regulation of gene expression, enzyme activities, and impact of genetic variation. Pharmacol Ther.

[32] Jana K, Bandyopadhyay T, Ganguly B (2018). Stereoselective Metabolism of omeprazole by cytochrome P450 2C19 and 3A4: Mechanistic Insights from DFT Study. J Phys Chem B.

[33] Saydam F, Değirmenci İ, Birdane A, Özdemir M, Ulus T, Özbayer C (2017). The CYP2C19*2 and CYP2C19*17 Polymorphisms play a vital role in clopidogrel responsiveness after percutaneous coronary intervention: a pharmacogenomics study. Basic Clin Pharmacol Toxicol.

[34] Kang P, Dalvie D, Smith E, Zhou S, Deese A, Nieman JA (2008). Bioactivation of flutamide metabolites by human liver microsomes. Drug Metab Dispos.

[35] Caccia S, Pasina L, Nobili A (2012). Critical appraisal of lurasidone in the management of schizophrenia. Neuropsychiatr Dis Treat.

[36] Shah S, Parmar B, Soniwala M, Chavda J (2016). Design, optimization, and evaluation of lurasidone hydrochloride nanocrystals. AAPS PharmSciTech.

[37] Drug Approval Package: Fanapt (iloperidone) NDA 022192. 2019. https://www.accessdata.fda.gov/drugsatfda_docs/nda/2009/022192s000TOC.cfm. Accessed 27 Nov 2019.

[38] Drug Approval Package:Latuda (lurasidone hydrochloride) NDA # 200603. 2019. https://www.accessdata.fda.gov/drugsatfda_docs/nda/2010/200603Orig1s000TOC.cfm. Accessed 27 Nov 2019.

[39] Francesco CD, Bickel MH (1977). Membrane lipids as intracellular binders of chlorpromazine and related drugs. Chem Biol Interact.

[40] MacIntyre AC, Cutler DJ (1988). The potential role of lysosomes in tissue distribution of weak bases. Biopharm Drug Dispos.

[41] Daniel WA (2003). Mechanisms of cellular distribution of psychotropic drugs. Significance for drug action and interactions. Prog Neuropsychopharmacol Biol Psychiatry.

[42] Kahma H, Filppula AM, Launiainen T, Viinamäki J, Neuvonen M, Evangelista EA (2019). Critical differences between enzyme sources in sensitivity to detect time-dependent inactivation of CYP2C8. Drug Metab Dispos.

[43] Zhang Z, Li Y, Shou M, Zhang Y, Ngui JS, Stearns RA (2004). Influence of different recombinant systems on the cooperativity exhibited by cytochrome P4503A4. Xenobiotica.

[44] Backes WL, Kelley RW (2003). Organization of multiple cytochrome P450s with NADPH-cytochrome P450 reductase in membranes. Pharmacol Ther.

[45] Brignac-Huber LM, Park JW, Reed JR, Backes WL (2016). Cytochrome P450 organization and function are modulated by endoplasmic reticulum phospholipid heterogeneity. Drug Metab Dispos.

